# 1958. Longitudinal SARS-COV-2 anti-spike antibody response in pregnant people with natural infection and variable vaccine uptake

**DOI:** 10.1093/ofid/ofac492.1584

**Published:** 2022-12-15

**Authors:** Sylvia M LaCourse, Morgan C Aurelio, Jaclyn N Escudero, Sascha R Ellington, Lauren B Zapata, Margaret C Snead, Krissy Yamamoto, Carol C Salerno, Alexander L Greninger, Alisa B Kachikis, Janet A Englund, Alison L Drake

**Affiliations:** University of Washington, Seattle, Washington; University of Washington, Seattle, Washington; University of Washington, Seattle, Washington; COVID-19 Response, Centers for Disease Control and Prevention, Atlanta, Georgia; COVID-19 Response, Centers for Disease Control and Prevention, Atlanta, Georgia; COVID-19 Response, Centers for Disease Control and Prevention, Atlanta, Georgia; University of Washington, Seattle, Washington; University of Washington, Seattle, Washington; University of Washington, Seattle, Washington; University of Washington, Seattle, Washington; Seattle Children's Hospital/ Univ. Washington, Seattle, Washington; University of Washington, Seattle, Washington

## Abstract

**Background:**

Natural SARS-CoV-2 infection results in anti-nucleocapsid (N) and anti-spike (S) antibody (Ab) development. Anti-S Ab response (conferred by infection and/or vaccination) is more closely associated with protection. We evaluated anti-N/S Ab responses in vaccinated (> 1 dose) and unvaccinated pregnant people with prior SAR-CoV-2 infection.

**Methods:**

During January 2021-March 2022, we enrolled participants with SARS-CoV-2 infection identified in pregnancy (26 via anti-N IgG+; 52 via prior RT-PCR+). Baseline, 1, 2, 3, 6, and 12 months, and delivery samples were tested for anti-N (index ≥ 1.4 positive) and anti-S (≥ 50 AU/mL positive) IgG Ab by Abbott Architect. Kaplan-Meier methods were used to measure Ab response duration.

**Results:**

Among 78 participants, 62 (79%) enrolled in pregnancy (median 27 weeks gestation), and 16 (21%) at delivery/postpartum (median 2 weeks); 34 (44%) had received ≥1 vaccine prior to initial Ab testing. At baseline, 59 (75%) participants had concordant anti-N/S positive results (median anti-N index 3.58 [IQR 2.01-5.82], median anti-S 5529 AU/ml [IQR 687-25000]). Anti-S IgG was higher (25000 vs 774, p< 0.001) among participants receiving ≥1 vaccine vs no vaccine, while anti-N IgG indices were similar.

Among 59 participants with initial anti-N IgG+ results, median time to anti-N IgG negative results was 31 weeks after first RT-PCR+ (median 17 weeks after first anti-N IgG+ result). Only 1 (unvaccinated) participant had an anti-S IgG negative result by 22 weeks after first RT-PCR+ result.

Among 30 participants with delivery samples (median 16 weeks after RT-PCR+, 12 weeks after baseline anti-N IgG+ samples), 15 (52%) remained anti-N IgG+; 29 (97%) remained anti-S IgG+. Anti-S IgG was higher (25000 vs 826 AU/ml, p< 0.001) among participants receiving ≥ 1 vaccine vs. no vaccine prior to delivery.

**Conclusion:**

Among pregnant persons with prior SARS-CoV-2 infection, duration of anti-S IgG response was longer than anti-N IgG irrespective of vaccine status; vaccination during pregnancy was associated with higher anti-S levels at baseline and delivery. While anti-S IgG were detectable for ≥ 6 months, longer term follow-up is needed to assess durability of hybrid immunity vs. infection alone and has implications for maternal and infant protection.

**Disclosures:**

**Sylvia M. LaCourse, MD, MPH**, Aurum Institute: Advisor/Consultant|Merck: Grant/Research Support **Alexander L. Greninger, MD, PhD**, Abbott: Contract Testing|Cepheid: Contract Testing|Gilead: Grant/Research Support|Gilead: Contract Testing|Hologic: Contract Testing|Merck: Grant/Research Support|Novavax: Contract Testing|Pfizer: Contract Testing **Alisa B. Kachikis, MD, MSc**, GSK: Advisor/Consultant|Merck: Grant/Research Support|Pfizer: Advisor/Consultant **Janet A. Englund, MD**, AstraZeneca: Advisor/Consultant|AstraZeneca: Grant/Research Support|GlaxoSmithKline: Grant/Research Support|Meissa Vaccines: Advisor/Consultant|Merck: Grant/Research Support|Pfizer: Grant/Research Support|Sanofi Pasteur: Advisor/Consultant **Alison L. Drake, PhD, MPH**, Merck: Grant/Research Support.

